# Effectiveness of Fibrin Sealant Patch in Reducing Drain Volume after Pelvic Lymph Node Dissection in Women with Gynecologic Malignancy

**DOI:** 10.1155/2017/3086857

**Published:** 2017-11-27

**Authors:** Hwa Cheong Kim, Chahien Choi, Woo Young Kim

**Affiliations:** Department of Obstetrics & Gynecology, Kangbuk Samsung Hospital, Sungkyunkwan University School of Medicine, Seoul, Republic of Korea

## Abstract

**Background:**

The goal of this study was to evaluate the effectiveness of fibrin sealant in decreasing postoperative lymphatic drainage in women after pelvic lymphadenectomy and/or para-aortic lymphadenectomy during gynecologic cancer surgery.

**Methods:**

This study is a retrospective case-control study. Forty-five patients who underwent staging surgery were enrolled. Twenty-seven patients were in the fibrin sealant group (group A) and 18 in the control group (group B). The two groups were compared for the total volume of drain, hospital stay, harvested lymph node, and incidence of asymptomatic lymphocele. Lymphocele formation was evaluated by computed tomography (CT) on 3 months after surgery.

**Results:**

There were no significant differences in patient demographics between group A and B with respect to age, BMI, and harvested lymph nodes. Patients who received fibrin sealants had reduced total volume of drainage from postoperative days 2 to 5 compared to the control group (group A versus group B: 994.819 ± 745.85 ml versus 1847.89 ± 1241.41 ml; *P* = 0.015). However no differences were observed in hospital stay (*P* = 0.282), duration of drain (*P* = 0.207), and incidence of asymptomatic lymphocele at 3 months (*P* = 0.126).

**Conclusion:**

The results of this study indicate that the application of fibrin sealants after pelvic and/or para-aortic lymphadenectomy may reduce lymphatic drainage in gynecologic malignancy.

## 1. Introduction

Pelvic and/or para-aortic lymph node dissection provides accurate staging information and helps guide clinical management in gynecologic cancer. Because gynecologic malignancies could metastasize through the lymphatics, it is important to evaluate the lymph node involvement to determine specific adjuvant treatment [1, 2]. However, complications related to the large amount of lymphatic flow after lymphadenectomy, such as lymphocele formation and lymphedema, have been reported in up to 60% of cases [3].

Lymphoceles are cyst-like structures that form due to excess lymphatic drainage in spaces created by node removal and vessel injury [4–6]. Lymphoceles can easily be diagnosed using computed tomography (CT), where they typically appear as hypodense fluid-filled structures with negative Hounsfield units (−18 HU), or on ultrasound (US), where they have a thin-walled cystic appearance with fluid content of varying echogenicity [7].

The incidence of lymphocele is unknown, and previously reported estimates ranged from 1.29 to 39.1% depending on the study method [8]. Most cases resolve spontaneously without intervention. Symptomatic lymphoceles commonly cause lower extremity edema, local infection, prolonged hospital stays, patient discomfort, and wound dehiscence [9]. Other complications associated with lymphoceles are pelvic infection, slow resumption of bowel movement, pelvic pain and/or heaviness, and lymphedema or leg swelling. In some circumstances, lymphoceles can cause life-threatening conditions that could require medical or surgical intervention. As such reducing lymphatic drainage and preventing lymphocele formation are important considerations for gynecologic physicians. Several methods have been introduced to reduce lymphatic drainage, including peritoneum-closing method, no-drainage placement approach, omentoplasty method, and leaving vagianal vault open method [4, 7]. Other methods for decreasing lymphatic flow were also described using energy-based methods such as Ultracision, Ligaclip, and bipolar method, combined with biological methods such as fibrin sealant [4, 10]. However, no consensus has been reached regarding the most effective method for preventing lymphocele formation.

Meanwhile, fibrin sealants such as TachoSil® [Takeda Austria GmbH, Linz, Austria] and Tisseel® [Baxter International Inc., Deerfield, IL, USA] have been reported as safe methods for achieving hemostasis during surgery [9, 11–14]. TachoSil and Tisseel action is based on the coagulation cascade and mechanical action as adhesives. They are typically used a supportive agent to create a surgical patch to improve hemostasis and promote tissue sealing [15].

Therefore, our hypothesis was that the use of fibrin sealant would be effective method for decreasing lymphatic drainage in gynecologic surgery with pelvic lymph node dissection. In this study we evaluated the feasibility of fibrin sealant for decreasing lymphatic drain, hospital stay, and incidence of lymphocele formation after surgery with pelvic and/or para-aortic lymphadenectomy.

## 2. Methods

From August 2013 to January 2017, we collected data from patients on whom fibrin sealants such as TachoSil and Tisseel were applied to the lymphadenectomy site during staging surgery for ovarian cancer, cervical cancer, or endometrial cancer at the Department of Obstetrics and Gynecology, Kangbuk Samsung Hospital.

All lymph nodes were handled in a standardized manner. Pelvic lymph nodes were fully dissected from bifurcation of common iliac vessels to deep circumflex iliac vein (near inguinal ligament). Para-aortic lymph node dissection was done up to the level of left renal vein by Thunderbeat. No cases of lymphadenectomy were performed with only lymph node sampling. We believed the sealing effect would eventually help prevent lymphatic extravasation. These were applied coincidently to the oozing areas of lymph node dissection. TachoSil was developed as a surgical patch for coagulation and sealing. Tisseel is a fibrin sealant used for coagulation with a formation of crosslink density increasing the effect of sealing. TachoSil is used for the broad areas of lymph node dissection. The surgical patch, such as TachoSil, has limited coverage for the narrow and steep areas due to its character of material. Because Tisseel is used as a liquid form, it was used for those areas that TachoSil could not cover.

For our control group, we retrospectively reviewed charts from patients who underwent the same operations without the use of fibrin sealants from February 2008 to January 2017.

Women with prior pelvic surgical histories, lymphatic system dysfunction, or problems with the immune system were excluded. Women with pelvic ascites or with peritoneal nodules were excluded, as pelvic ascites can be confused with lymphatic drainage.

A total of 45 patients were enrolled in the study: 27 patients were in the fibrin sealant group (group A) and 18 in the control group (group B). Drain volume was recorded daily using 200 cc or 400 cc Hemovac with full negative pressure, and daily drainage and length of hospital stay were compared between the two groups.

Patients were evaluated by CT on postoperative day (POD) 7 as well as 1 and 3 months after surgery.

The primary outcome was to evaluate the effectiveness of fibrin sealants in reducing the total volume of drain. Total volume of lymphatic drain was defined as the sum of daily drainage from POD 2 to 5. The secondary outcome was to evaluate the incidence of lymphocele. Lymphocele was defined as presence of hypodense contents with negative Hounsfield units (−18 HU) by CT [7]. If patients had symptomatic lymphocele, interventions such as percutaneous drainage have been used and followed by sclerotherapy for prevention of symptomatic lymphocele recurrence.

### 2.1. Statistical Analysis

All statistical analyses were performed using IBM SPSS software (ver 24.0; SPSS Inc., Armonk, NY, USA). Data are reported as mean ± standard deviation (SD) or number (%), unless otherwise indicated. Categorical variables were assessed using Fisher's extract test or the chi-square test. The *t*-test was used to assess group differences for continuous variables. A *P* value of less than 0.05 was considered statistically significant.

## 3. Result

In this study, there were no significant differences in patient demographics between group A and B with respect to age, BMI, previous treatment, harvested lymph nodes, and past medical history (Table 1).

In group A, seventeen patients (62.96%) underwent laparoscopic surgery, and 10 (37.04%) underwent open surgery. In group B, fourteen patients (77.78%) underwent laparoscopic surgery, and 4 (22.2%) underwent open surgery. The difference in type of surgery between the two groups was not statistically significant (*P* = 0.610).

Final pathology between the two groups was also not significantly different (*P* = 0.088). In group A, seven patients (25.9%) were diagnosed with early stage ovarian cancer, 9 (33.3%) with cervical cancer, and 11 (40.7%) with endometrial cancer. In group B, two patients (11.1%) were diagnosed with early stage ovarian cancer, 10 (66.7%) with cervical cancer, and 6 (22.2%) with endometrial cancer.

The number of harvested lymph nodes was 22.07 ± 8.68 in group A and 27.61 ± 12.70 in group B, which was also not statistically significant (*P* = 0.089).

The daily drain volume of group A was statistically smaller than that of group B at POD 2 (292.63 ± 189.34 ml versus 644.39 ± 519.89 ml; *P* = 0.012) and POD 3 (286.22 ± 222.87 ml versus 584.00 ± 511.09 ml; *P* = 0.030) (Table 2).

The average day of drain removal was POD 5.7 ± 2.32 in group A and POD 7.0 ± 3.80 in group B (*P* = 0.207). Although the difference was not significant, drain removal occurred earlier in group A than in group B.

Finally, there was also no significant difference in the incidence of lymphocele between the two groups 3 months after surgery (11.1% versus 33.3% *P* = 0.126) (Table 2).

## 4. Discussion

Excessive lymphatic drainage in women with gynecologic malignancies is a complication that often requires additional interventions and delays the initiation of adjuvant treatment [12, 13]. Although several methods such as peritoneal repair, lymphangiography, and the use of fibrin sealants are currently being used after pelvic and/or para-aortic lymphadenectomy to prevent problems associated with lymphatic drainage [5, 16, 17], there is currently no standardized approach.

Fibrin sealants have recently started to get attention. Promising results using fibrin sealants such as TachoSil after groin dissection or pelvic lymph node dissection for urologic cancer suggest that the application of fibrin sealants could be similarly effective in gynecologic cases [11]. Benevento et al. reported that fibrin sealants were able to reduce the output of serum from the axilla in patients who underwent axillary lymphadenectomy for breast cancer [9]. Fibrin sealants help to augment the final stage of coagulation when fibrinogen is converted into stable fibrinogen clot [18]. Fibrin formation not only controls bleeding, but also creates an adhesive barrier. It could be expected that the fibrin sealant's hemostatic and adhesive properties could help reduce lymphatic drainage by sealing damaged lymphatic vessels via a less traumatic mechanism [18].

Recently, Kim et al. reported in a randomized controlled study that hemostatic sealant FloSeal® was effective in preventing lymphocele and reducing lymphatic drainage in patients who had pelvic and para-aortic lymphadenectomy for gynecologic cancer [19]. However the study by Kim et al. also included eight cases of advanced stage ovarian cancer with ascites. This shows that this study had limitation of differentiating lymphatic drainage from ascites. This difficulty makes their lymphatic drainage result less reliable. Our study excluded patients with advanced stage ovarian cancer, thereby preventing confusion between lymphatic drainage and ascites.

In the present study, using fibrin sealants was effective in reducing the total volume of lymphatic drain after pelvic and/or para-aortic lymphadenectomy, regardless of pathologic subtype or surgery type. Total drain volume was defined as sum of daily drain output from POD 2 to 5. The total volume of drainage was statistically different between group A and group B (group A versus group B: 994.819 ± 745.85 ml versus 1847.89 ± 1241.41 ml; *P* = 0.015). However, there was no significant difference regarding lymphatic drainage at POD 1 between group A and group B. We did not include this data point due to the practice of using intraoperative irrigation fluids, which likely accounts for a large fraction of the fluid drained in the immediate postoperative state. However, it is worth mentioning that the difference between that two groups remained statistically significant even when POD 1 output was included (group A versus group B: 1244.15 ± 828.20 ml versus 2327.61 ± 1527.21 ml; *P* = 0.011).

Previous studies have reported that fibrin sealants not only reduce the output of serum from the axilla in women with breast cancer, but also reduce length of hospital stay [9, 20, 21]. In many previous reports regarding breast cancer surgery with axillary lymph node dissection, fibrin sealants proved to decrease lymphatic fluid and reduce length of hospital stay [9, 12]. Felsingerova et al. showed that large volume of lymphatic flow would prolong hospital stay and increase patient discomfort after staging surgery with pelvic lymph node dissection [22]. However, in our study, there was no statistically significant difference in hospital stay between the two groups, likely because patients in our country are usually not discharged until pathology is confirmed. In Korea, most of the patients are taken care of in hospitals until a full recovery of wound and the first chemotherapy due to the low personal expenses with most of the expenses covered by the public health insurance. Therefor the duration of hospital stay is more associated with the date of pathologic confirmation or the day of total removal of stitch and first treatment of chemotherapy.

Although many previous studies have described the effects of fibrin sealants on the prevention of lymphocele formation (19.2% of patients in TachoSil group versus 51.7% in the control group) after laparoscopic staging surgery with pelvic lymph node dissection [13], there have also been studies with negative results. Achouri et al. reported that fibrin sealants were not effective in preventing lymphocele formation after lymphadenectomy in pelvic gynecologic malignancy [4].

Lymphatic injury is the main causative factor of the formation of lymphocele. However, a number of variables should be taken into consideration when comparing the incidence of lymphocele including the method of lymphocele detection, follow-up interval, use of energy source, surgical approach, body mass index, and the number of harvested lymph nodes [13]. Many literature sources have reported on the above factors to evaluate the correlation with incidences of lymphocele [4, 6]. Köhler et al. reported that 33% patients developed infected lymphoceles on the side of the discolored lymph node due to tattoo [23]. In this study, there was no one with tattoos on the extremities. In our study, the incidence of lymphocele was not significantly different between the fibrin sealant and control groups. There was only one case that showed complication at 1 month after surgery in group B; the patient was found to have tenderness of the abdomen, which was a small cyst of less than 3 cm that could disappear with only 5 days of antibiotic treatment. Except for that case, there were no symptomatic lymphoceles and no intervention was needed for 3 months after surgery.

Our study does have a number of limitations. First, it had a relatively small number of study participants. Although the duration of drain seemed to decrease, we could not draw a conclusion about the usefulness of fibrin sealants due to statistical insignificance. We think larger sample size is needed to validate our result. Second, the period of data collection was different between the two groups. Despite these limitations, our study shows that fibrin sealant may be of help to reduce the lymphatic drainage after pelvic and/or para-aortic lymphadenectomy in gynecologic malignancy.

In our study, although we could not prove statistical significance, the group that used fibrin sealants had shorter duration of drainage use. The removal of drainage not only can lessen the burden of patients physically and mentally but also can be beneficial for their quality of life. These results support the conclusion that fibrin sealant such as TachoSil and Tisseel may be used to control lymphatic flow after pelvic and/or para-aortic lymphadenectomy. However, a large-scale randomized trial will be needed to validate the results of this study.

## 5. Conclusion

The results of this study indicate that the application of fibrin sealants after pelvic and/or para-aortic lymphadenectomy may reduce lymphatic drainage in gynecologic malignancy.

## Figures and Tables

**Table 1:** Characteristics of patients.

Characteristic	Group A (*n* = 27)	Group B (*n* = 18)	*P* value
Age (yrs)	58.07 ± 10.30	53.56 ± 12.41	0.191
BMI^1^ (kg/m^2^)	24.81 ± 4.69	23.16 ± 2.88	0.153
LN^2^ positive (%)			0.098
LN (+)	2 (7.4%)	5 (27.8%)	
LN (−)	25 (92.6%)	13 (72.2%)	
Number of removed LN	22.07 ± 8.68	27.61 ± 12.70	0.089
Diagnosis			
Ovary cancer	7 (25.9%)	2 (11.1%)	
BPLND^3^	5	2	
BPLND + BPALND^4^	2	0	
Endometrial cancer	11 (40.7%)	6 (22.2%)	
BPLND	9	5	
BPLND + BPALND	2	1	
Cervix cancer	9 (33.3%)	10 (66.7%)	
BPLND	5	6	
BPLND + BPALND	4	4	

^1^BMI: body mass index; ^2^LN: lymph node; data are expressed as the means ± standard deviation, medians (range), or frequencies (percentages), as appropriate; ^3^BPLND: both pelvic lymph node dissection; ^4^BPALND: both para-aortic lymph node dissection.

**Table 2:** Volume of lymphatic drainage and incidence of lymphocele.

Outcome	Group A (*n* = 27)	Group B (*n* = 18)	*P* value
Mean total vol.^1^ drained^2^ (ml)			
Drain vol. day 1	339.11 ± 236.10	509.72 ± 441.77	0.099
Drain vol. day 2	292.63 ± 189.34	644.39 ± 519.89	0.012
Drain vol. day 3	286.22 ± 222.87	584.00 ± 511.09	0.030
Drain vol. day 4	207.30 ± 218.16	305.89 ± 358.60	0.306
Drain vol. day 5	208.67 ± 265.50	313.61 ± 369.87	0.274
Total volume of drainage	994.819 ± 745.85	1847.89 ± 1241.41	0.015
(from day 2 to 5)			
Accumulation	1244.15 ± 828.20	2327.61 ± 1527.21	0.011
(from day 1 to 5)			
Mean duration of drain (day)	5.7 ± 2.32	7.0 ± 3.80	0.207
Mean length of stay (day)	10.89 ± 3.71	12.72 ± 6.40	0.282
Discharge with drain	0	0	
(people)			
Complication	0	0	0.400
Yes	0 (0%)	1 (5.6%)	
No	27 (100%)	17 (94.4%)	
Incidence of lymphocele^3^			
1 month	3/27 (11.1%)	3/18 (16.7%)	0.670
3 months	3/27 (11.1%)	6/18 (33.3%)	0.126

^1^Vol.: volume; ^2^drain: drain from Hemovac for 24 hr; ^3^incidence of lymphocele: hypodense content with negative −18 Hounsfield unit ^*∗*^(HU) by computed tomography images; data are expressed as the means ± standard deviation, medians (range), or frequencies (percentages), as appropriate.
